# Analgesic and Neuroprotective Effects of Electroacupuncture in a Dental Pulp Injury Model—A Basic Research

**DOI:** 10.3390/ijms21072628

**Published:** 2020-04-09

**Authors:** Sharmely Sharon Ballon Romero, Yu-Chen Lee, Lih-Jyh Fuh, Hsin-Yi Chung, Shih-Ya Hung, Yi-Hung Chen

**Affiliations:** 1 Graduate Institute of Acupuncture Science, China Medical University, Taichung 40402, Taiwan; Sharmely@hotmail.com (S.S.B.R.); d5167@mail.cmuh.org.tw (Y.-C.L.); mowmow0202@gmail.com (H.-Y.C.); shihyahung@mail.cmu.edu.tw (S.-Y.H.); 2 Department of Acupuncture, China Medical University Hospital, Taichung 40447, Taiwan; 3 Chinese Medicine Research Center, China Medical University, Taichung 40402, Taiwan; 4 School of Dentistry, College of Dentistry, China Medical University; Taichung 40402, Taiwan; ljfuh@mail.cmu.edu.tw; 5 Department of Medical Research, China Medical University Hospital, Taichung 40447, Taiwan; 6 Department of Photonics and Communication Engineering, Asia University, Taichung 41354, Taiwan; 7 Brain Disease Research Center, China Medical University Hospital, Taichung 40447, Taiwan

**Keywords:** dental pulp injury, inflammation, microglia, astrocytes, electroacupuncture

## Abstract

Irreversible pulpitis is an extremely painful condition and its consequence in the central nervous system (CNS) remains unclear. A mouse model of dental pulp injury (DPI) resembles the irreversible pulpitis profile in humans. This study sought to determine whether pain induced by DPI activates microglia and astrocytes in the trigeminal subnucleus caudalis (Vc), as well as increases levels of proinflammatory cytokines, and whether electroacupuncture (EA) can be a potential analgesic and neuroprotective therapy following DPI. Pain behavior was measured via head-withdrawal threshold (HWT) and burrowing behavior at days 1, 3, 7, 14 and 21 after DPI. A marked decrease in HWT and burrowing activity was observed from day 1 to 14 after DPI and no changes were seen on day 21. Microglial and astrocytes activation; along with high cytokine (TNFα, IL-1β, and IL-6) levels, were observed in the Vc at 21 days after DPI. These effects were attenuated by verum (local and distal) EA, as well as oral ibuprofen administration. The results suggest that DPI-induced pain and glial activations in the Vc and EA exert analgesic efficacy at both local and distal acupoints. Furthermore, verum (local and distal) EA might be associated with the modulations of microglial and astrocytes activation.

## 1. Introduction

Pain associated with inflammation of the dental pulp, pulpitis, is anecdotally described as “*the highest level possible*” [1] and is one of the most common oral diseases affecting humans worldwide and is majorly contributed by bacterial infection due to the development of caries [2]. A mild pulpal inflammation is carried out by reversible pulpitis that resolves and returns to normal once the etiology is removed. In irreversible pulpitis, dental pulp damage is beyond self-repair and untreated-pulp gradually becomes necrotic [3]. Irreversible pulpitis is most often caused by dental caries, a cracked tooth, or by trauma; any tooth may be affected and the condition can develop in any age group [4]. Previous studies have reported changes in the morphology and neurochemical characteristics of the intradental nerve in response to injury [5,6]. Although, more evidence is needed to understand better the way in which the local region interacts with the central nervous system (CNS) after injury.

Microglia are considered the main immune defense and account for approximately 10%–20% of all cells in the CNS. In response to injury, microglia undergo a morphological shift from a resting to an activated state [7]. These changes are usually accompanied by variations in gene signaling and expression, as well as the release of reactive nitrogen, oxygen species and proinflammatory cytokines, such as tumor necrosis factor-alpha (TNF-α), interleukin-1β (IL-1β) and IL-6 [8]. In turn, these cytokines activate astrocytes that support microglial functioning, including phagocytosis and activation of macrophages [9]. Interactions between microglia and cytokines are necessary for the activation and proliferation of the astrocytic response and microglial recruitment [8,10]. The central mechanisms of orofacial pain were associated with microglial activation as in an experimental model of dental pulp injury (DPI), in which pulp exposure caused a significant microglial activation in the trigeminal subnucleus caudalis (Vc) [11]. However, the interaction between microglia and astrocytes after pulp exposure has not yet been explored. 

Conventional analgesic treatment for pulpitis consists of nonsteroidal anti-inflammatory drugs (NSAIDs) such as ibuprofen [12]. Oral administration of NSAIDs pre- or post-operatively reduce pain and achieve better patient outcomes although are associated with multiple side effects including nausea, vomiting and gastrointestinal alterations [13]. Moreover, managing pain in dentistry is complicated and more effective alternatives alone or in combination are needed. In accordance, acupuncture is an ancient technique frequently used to relieve pain, offering fewer side effects with simple and safe procedure [14]. In 2017, a randomized double-blind clinical trial involving patients with symptomatic irreversible pulpitis demonstrated that classical acupuncture provided more effective pain relief (faster and prolonged) over 48 h compared with ibuprofen [15]. Furthermore, the World Health Organization supports acupuncture for its efficacy in several symptoms, diseases, or conditions and facial pain (including the craniomandibular disorders) [16]. 

This novel study sought to determine for the first time whether pain induced by DPI activates microglia and astrocytes in the trigeminal subnucleus caudalis (Vc) of mice as well as increases levels of proinflammatory cytokines and whether electroacupuncture (EA) can be a potent analgesic and neuroprotective therapy following DPI.

## 2. Results

### 2.1. Analgesic Effect of Electroacupuncture on DPI-Induced Mechanical Allodynia

**Figure 1:**
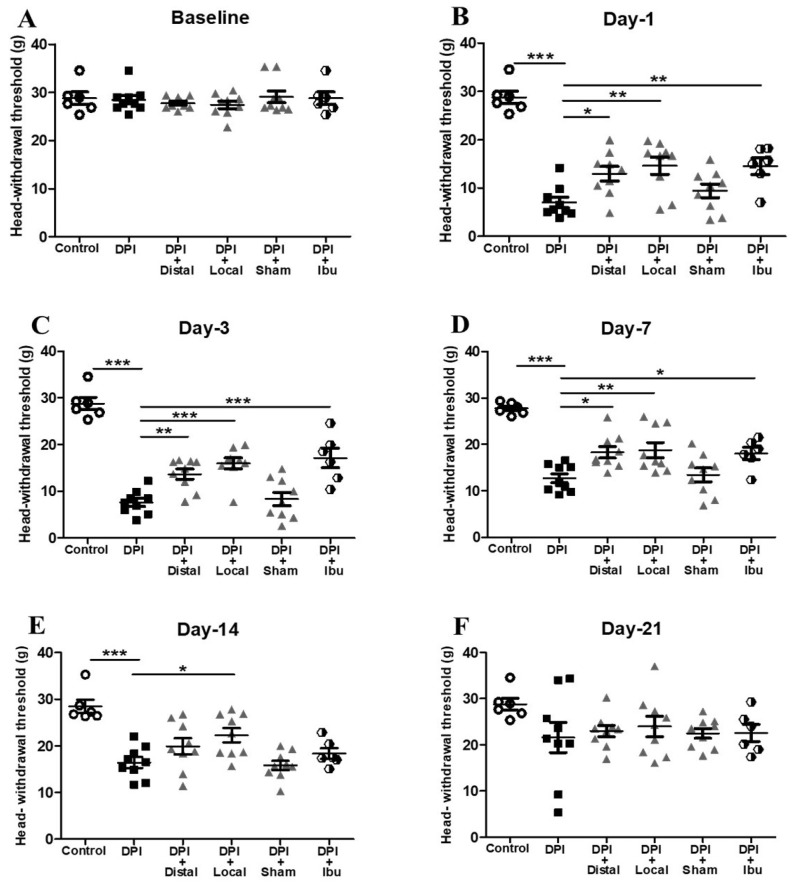
Verum (local and distal) electroacupuncture (EA) and ibuprofen alleviated mechanical allodynia following dental pulp injury (DPI). (**A**) Baseline measurements. (**B**) On day 1, mice in the DPI group exhibited a robust sign of mechanical allodynia when compared with controls. These effects were significantly attenuated by verum (local and distal) EA, as well as by ibuprofen. (**C**) On day 3, verum (local and distal) EA, along with ibuprofen, demonstrated good analgesic effects compared with the DPI group. (**D**) On day 7, the local EA was associated with a higher nociceptive threshold than either distal EA or ibuprofen. (**E**) On day 14, only local EA significantly showed analgesic effectiveness (**F**) On day 21, there was no difference in pain-related behaviors among all studied groups. Sham EA failed to demonstrate any significant effects upon mechanical allodynia on any of the testing days. Between-group comparisons for each group were performed by ANOVA, followed by Dunnett’s test (* *p* < 0.05, ** *p* < 0.01, *** *p* < 0.001, versus DPI group; *n* = 9 in each group).

DPI is a characteristic orofacial pain model and has been reported to evoke pain-like and anxiety behaviors, as previously described [17]. In order to follow the previous methods, we have performed a unilateral behavioral assessment in the surrounding skin area of the compromised tooth. One day prior to DPI all groups showed similar responses in von Frey testing, with a maximum nociceptive threshold of around 30 g, as a baseline (Figure 1A). Mice that underwent only DPI exhibited the development of mechanical allodynia. A persistent and significant mechanical allodynia was observed from day 1 to 14 after pulp exposure in the DPI group, but no mechanical allodynia was seen at day 21 (Figure 1B–E). To investigate that DPI-induced pain, we administered ibuprofen as analgesic treatment. Interestingly, on days 1, 3 and 7, mechanical allodynia was greatly ameliorated by ibuprofen (30 mg/kg) orally administrated the first three days after DPI, followed by a consecutive single dose every other day for 21 days and 30 min prior to behavior testing days (Figure 1B–D). However, on days 14 and 21, there were no significant differences between the ibuprofen treated group and the DPI group (Figure 1E,F). To further address the role of allodynia after DPI and evaluate the analgesic effect of EA, two pairs of verum acupoints (local and distal), which are commonly used to treat orofacial pain, and one pair of sham acupoints were selected [15,18,19]. The EA sessions followed the same schedule as ibuprofen treatment. The results showed that consecutive sessions of verum (local and distal) EA, reversed the mechanical allodynia at days 1, 3 and 7 (Figure 1B–D). However, the analgesic effect of EA only at local acupoints demonstrated significance efficacy at day 14 (Figure 1E). At all assessed time points, sham EA had no significant effect on the nociceptive threshold when compared to the DPI group (Figure 1B–E). On day 21, the nociceptive threshold increased and returned to normal and there were no significant differences in pain-related behaviors between groups (Figure 1F). Overall, these results suggested that verum (local and distal) EA had a clearly positive effect on mechanical allodynia comparable to ibuprofen in the DPI mice model (Table S1).

### 2.2. Electroacupuncture Attenuates Changes in Burrowing Behavior after DPI

**Figure 2:**
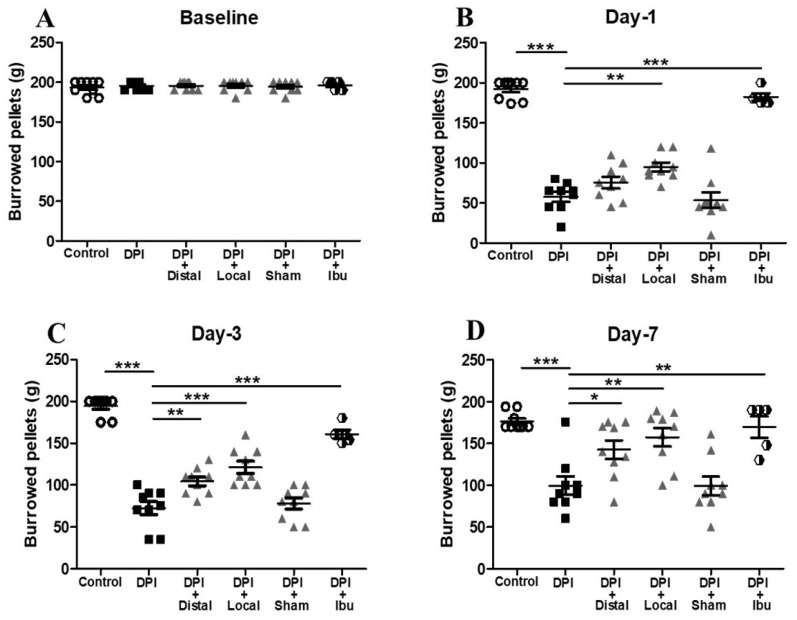

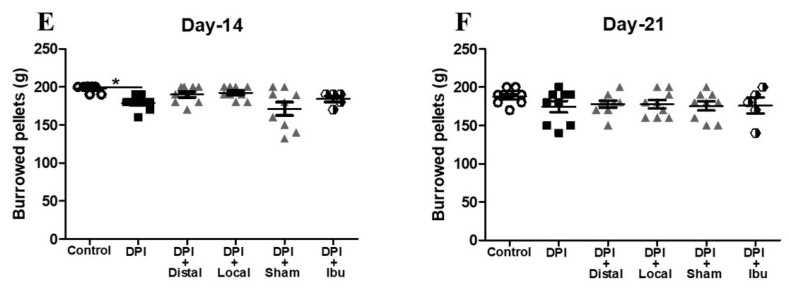
DPI-induced suppression of burrowing activity is reversed by verum (local and distal) EA and ibuprofen treatment. Baseline measurements. (**B**) On day 1 after DPI, burrowing activity only in the DPI group was significantly reduced when compared to controls; Local EA and ibuprofen treatment showed significant analgesic effects. (**C**,**D**) On days 3 and 7, respectively, mice underwent verum (local and distal) EA and ibuprofen treatment exhibited a significant increment in burrowing activity when compared with the DPI group. (**E**) On day 14, burrowing activity in the DPI group remained lower when compared with the other studied groups. (**F**) On day 21, comparable burrowing activity in all studied groups of mice. Sham EA failed to evidence any significant effects in burrowing activity on any of the testing days. Between-group comparisons for each group were performed by ANOVA, followed by Dunnett’s test (* *p* < 0.05, ** *p* < 0.01, *** *p* < 0.001, versus DPI group; *n* = 9 in each group).

To further evaluate the impact of DPI-induced pain in rodents, we assessed the instinctive and remarkably motivated burrowing behavior as a “well-being” indicator. At baseline, all 5 groups of mice exhibited similar burrowing activity, each group burrowed an approximate total of 200 g of pellets (Figure 2A). Consistent with our previous von Frey results, mice under only DPI, showed a significant decrease in the cumulative weight of pellets burrowed from day 1 to 14, in comparison to the no significant changes observed at day 21 (Figure 2B–F). To probe that potential of DPI-induced pain, we administered NSAID treatment (i.e., ibuprofen) as previously described. Our data demonstrated that oral ibuprofen doses of 30 mg/kg in mice produced a marked antinociceptive effects in the burrowing tests with a significant increment of pellets burrowed on days 1, 3 and 7 compared to DPI group (Figure 2B–D). Based on the von Frey test results, we selected the same group of acupoints at the same schedule as previously described. Successive sessions of verum (local and distal) EA increased significantly the spontaneous burrowing activity in the mice on days 1, 3 and 7 when compared to DPI group (Figure 2B–D). However, sham EA failed to produce antinociception in the same testing days. Interestingly, all groups of mice underwent DPI and additional treatment (EA or Ibuprofen) demonstrated similar burrowing activity on days 14 and 21 with no significant differences among them (Figure 2E,F). In addition, the burrowing results suggested, that EA and ibuprofen offer analgesic effects following DPI. Although, ibuprofen showed superior results when compared to verum (local and distal) EA (Table S2). 

### 2.3. Microglial and Astrocytes Activation in the Trigeminal Subnucleus Caudalis (Vc) after DPI

**Figure 3:**
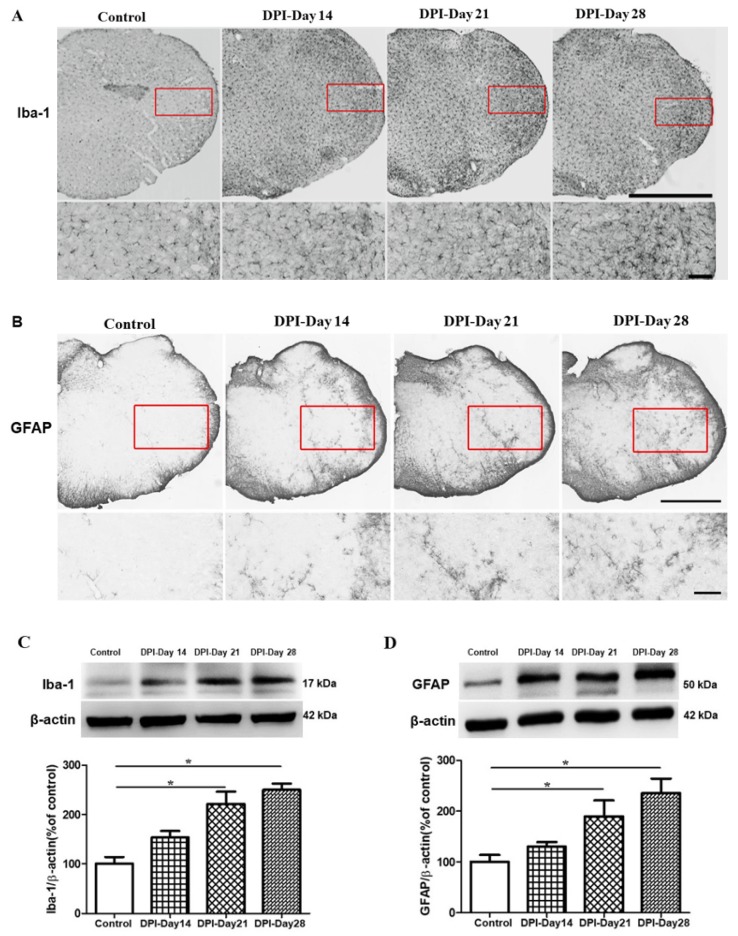
Microglial and astrocytes activation on Day 21 and persisted through Day 28 after DPI. (**A**,**B**) Representative photomicrographs of Iba-1 and GFAP expression respectively, in 4 groups of mice: controls, DPI-Day 14, DPI-Day 21 and DPI-Day 28. (**C**,**D**) Western blot analysis revealed no significant differences in Iba-1 and GFAP protein levels between the DPI-Day 14 group and controls, whereas Iba-1 and GFAP protein levels were significantly increased on days 21 and 28 when compared with the control group. Histogram showing incremental increases in Iba1 and GFAP levels after DPI. Between-group comparisons for each group were performed by ANOVA, followed by Dunnett’s test (* *p* < 0.05, versus control group; *n* = 8 in each group). Scale bars = 1 mm (upper panel), 100 μm (lower panel) in (**A**) and 1 mm (upper panel), 250 μm (lower panel) in (**B**).

We investigated the principal response to pain within the trigeminal system after DPI. Since the brainstem (i.e., trigeminal subnucleus caudalis) is a critical site in the neural pathways that mediate noxious stimulation from craniofacial tissues [20]. In our immunohistochemistry study, the morphological changes in microglia were identified by using the Iba-1 antibody, and for astrocytes GFAP as the prototypical marker. Following DPI, microglia and astrocyte shape changes were observed on day 14, which peaked on day 21 and persisted through day 28 (Figure 3A,B). These results supported the notion that DPI was involved in microglial and astrocytes activation in the Vc. In addition to immunohistochemistry and in order to confirm glial activation, levels of Iba-1 and GFAP proteins were determined by Western blotting. To investigate whether DPI-induced microglia and astrocytes as a time-dependent manner, we examined the levels Iba-1 and GFAP at all 3 assessment times. The expression of Iba-1 and GFAP were significantly enhanced in DPI-Day 21 and persisted through DPI-Day 28 when compared to controls (*p* < 0.05). However, there were no statistically significant differences between the DPI-14 days and the control group. These findings suggested that microglia and astrocyte activation occur in a time-dependent manner in the DPI mice model (Figure 3C,D).

### 2.4. EA at Verum (Local and Distal) Acupoints Inhibits Microglial and Astrocytes Activation Induced by DPI

**Figure 4:**
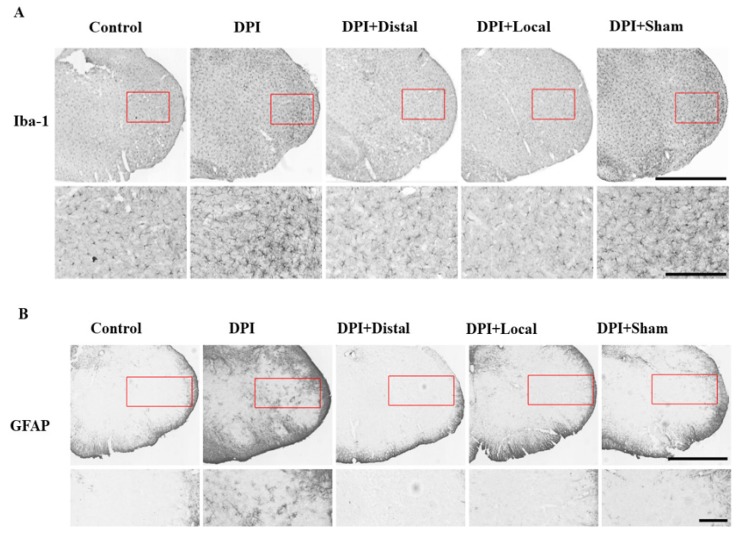

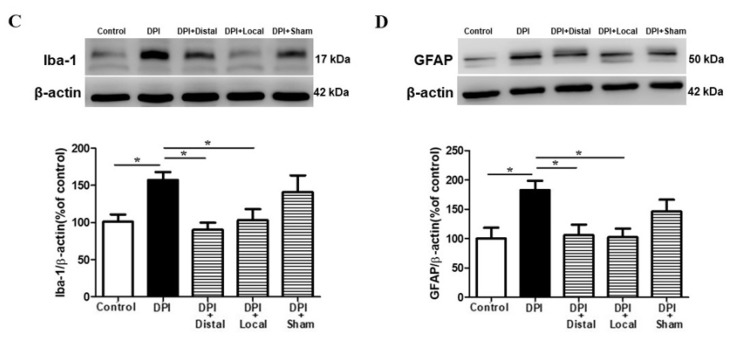
Verum (local and distal) EA inhibits DPI-induced microglial and astrocytes activation in the subnucleus caudalis (Vc). (**A**,**B**) Photomicrographs of Iba-1 and GFAP immunoreactivity, respectively, increased on day 21 after DPI in the Vc compared to controls, whereas verum (local and distal) EA downregulated the expression of microglia and astrocytes, while sham EA did not have such effect. (**C**,**D**) Illustrative Western blot bands, histogram and data summary depicting Iba-1 and GFAP expression, which are microglia and astrocytes markers respectively. Between-group comparisons for each group were performed by ANOVA, followed by Dunnett’s test (* *p* < 0.05, versus DPI group; *n* = 9 in each group). Scale bars = 1 mm (upper panel), 250 μm (lower panel) (**A**,**B**).

There is increasing evidence that strongly supports the involvement of microglial and astrocytes activation in orofacial pain [21]. To evaluate whether EA at 2 Hz, affects the activation of microglia and astrocytes, we investigated the levels of Iba-1 and GFAP proteins in the Vc. Our following results were based on the peak activation phase, at 21 days after DPI, as consistent with our findings and a previous report [11]. Immunohistochemistry staining was performed to explore the microglial and astrocytes morphological changes in the Vc after DPI. Microglia and astrocytes morphologies changed dramatically on day 21 after DPI, microglia immunoreactivity showed amoeboid shapes with short and thick processes, while astrocytes appeared hypertrophic with thick processes in the DPI group when compared with controls. In the groups of verum (local and distal) EA, microglia and astrocytes did not demonstrate a remarkable sign of activation showing habitual small cell bodies with thin processes as in the resting morphologies. In the sham EA group, microglia and astrocytes morphology exhibit signs of activation similar to the DPI group (Figure 4A,B). These results supported the hypothesis that EA may inhibit glial activation in Vc after DPI. In addition to immunohistochemistry assessment, Western blotting was performed to evaluate levels of Iba-1 and GFAP proteins. The expression of Iba-1 and GFAP proteins were elevated in the Vc of DPI mice when compared to control group (*p* < 0.05). Verum (local and distal) EA, downregulated the expression of microglia and astrocytes when compared to the DPI group (*p* < 0.05). Furthermore, sham EA show similarly high levels of Iba-1 and GFAP proteins as in the DPI group (Figure 4C,D). These results suggested that verum (local and distal) EA may inhibit microglial and astrocytes activation in Vc following DPI.

### 2.5. Verum EA Reduces DPI-Induced Production of Pro-Inflammatory Cytokines

**Figure 5:**
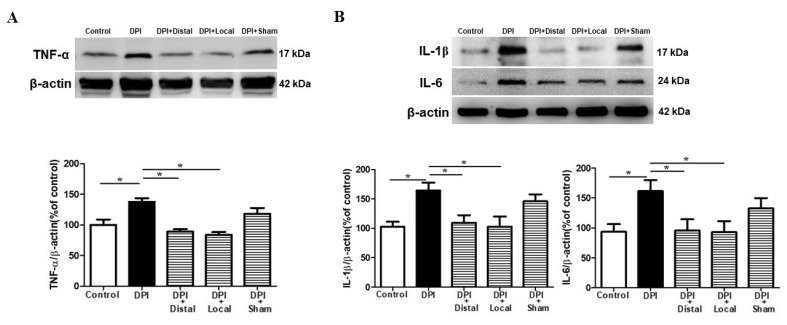
Verum (local and distal) EA inhibits DPI-induced production of pro-inflammatory factors in the Vc. All measured cytokines were markedly increased in the DPI group when compared with controls. (**A**) Verum (local and distal) EA, but not sham EA, reduced the production of pro-inflammatory cytokine TNF-α. (**B**) Verum (local and distal) EA but not sham EA, suppressed IL-1β and IL-6 production in the Vc. Illustrative Western blot bands, histogram and data summary depicting TNF-α, IL-1β and IL-6 expression. Between-group comparisons for each group were performed by ANOVA, followed by Dunnett’s test (* *p* < 0.05, versus DPI group; *n* = 7 in each group).

Tumor necrosis factor-α (TNF-α), interleukin-1β (IL-1β), interleukin-6 (IL-6) are pro-inflammatory cytokines that stimulate glial cell as an inflammatory response. EA has been reported to regulate the production of pro-inflammatory cytokines in neuropathic pain models [22]. The expression of cytokines (TNF-α, IL-1β and IL-6) in the Vc were verified by Western blotting 21 days after DPI, consistent with the activation phase of microglia and astrocytes as previously reported in Figure 3 and Figure 4. The DPI group shows high levels of TNF-α, IL-1β, and IL-6, when compared to the control group (*p* < 0.05). However, EA treatment (3-weeks sessions, every other day, 11 sessions in total) at verum (local and distal) acupoints inhibited the production of TNF-α, IL-1β, and IL-6 (*p* < 0.05) when compared to DPI group (Figure 5A,B). 

There were no significant differences between DPI and the sham EA group. These findings suggested that DPI-induced high levels of TNF-α, IL-1β, and IL-6, while verum (local and distal) EA inhibited proinflammatory cytokines production in the Vc of the brainstem (Figure 4A,B). Overall, these results suggest that verum (local and distal) EA was involved in the downregulation of activated microglial and astrocytes after DPI.

### 2.6. Ibuprofen Prevents DPI-Induced Changes in Microglial, Astrocyte and Cytokine Expression in the Vc

**Figure 6:**
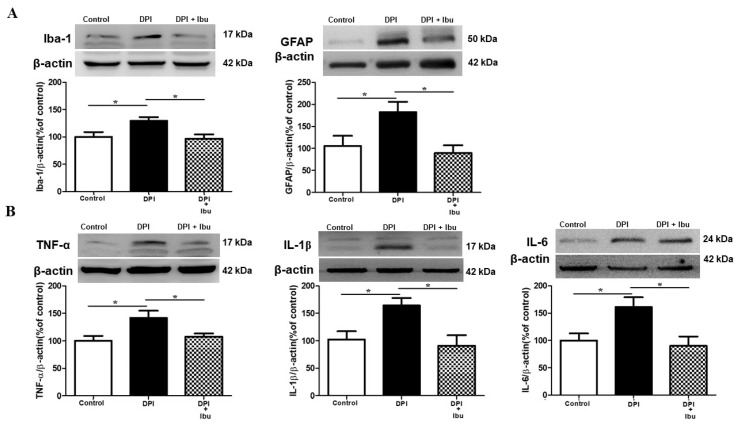
Ibuprofen restrains DPI-induced changes in glial and cytokine expression in the Vc. (**A**,**B**) Ibuprofen treatment inhibited high levels of microglial and astrocytes markers (Iba1 and GFAP, respectively), as well as reduced the production of pro-inflammatory cytokine TNF-α, IL-1β, and IL-6 expression following DPI. Between-group comparisons for each group were performed by ANOVA, followed by Dunnett’s test (* *p* < 0.05, versus DPI group; *n* = 6 in each group).

In order to explore the effects of ibuprofen treatment on microglia, astrocytes and proinflammatory cytokines (TNF-α, IL-1β, and IL-6) in a DPI model, Western blotting analysis were performed. The Vc area of the brainstem was collected at 21 days after DPI; ibuprofen treatment was orally administrated on a daily basis for the first three days after DPI, followed by a consecutive administration every other day. The DPI group shows the overexpression of neuroinflammatory markers. Ibuprofen decreased high levels of Iba-1 and GFAP and inhibited the production of TNF-α, IL-1β, and IL-6 in the Vc induced by DPI (*p* < 0.05), (Figure 6A,B). Moreover, these results strongly suggested the high levels of Iba-1, GFAP, TNF-α, IL-1β and IL-6 in the Vc of the brainstem following DPI. 

## 3. Discussion

To the best of our knowledge, our study describes for the first time the neuroinflammatory profile of the DPI mice model, involving microglia and astrocyte activation in a time-dependent manner. In general line with this conclusion, EA treatment exerts analgesic effect at verum acupoints, suppressing mechanical allodynia of the maxillary nerve after DPI and diminishes orofacial pain disability as indicated by burrowing activity, which can be a valuable tool to assess the global impact of dental pain on the mice well-being. Moreover, verum EA as well as ibuprofen had a significant neuroprotective effect on the DPI-induced glial activation and inhibited the production of pro-inflammatory cytokine in Vc. 

### 3.1. Behavioral Measurements in the DPI Model

Previous studies have demonstrated that DPI affects negatively innate behaviors, exploratory behaviors, mechanical withdrawal thresholds, which are elucidated as signs of pain [17,23,24]. The present study revealed that DPI (injury of the maxillary nerve) affected rodent’s daily activities, causing a marked reduction in burrowing activity, and this finding is consistent with previous related work [25]. Moreover, researchers have reported no significant difference in frequency and duration on grooming behavior after DPI on day 14 [17]. In concordance with this result, we noted no significant difference between groups in head withdrawal thresholds and burrowing behavior on day 21 after DPI. Furthermore, in the present study, we have demonstrated that verum (local and distal) EA, which may induce analgesic effects by stimulating the Aβ and Aδ-fibers involving multiple possible mechanisms [25], produced a significant increase in withdrawal threshold that evoke an anti-nocifensive response after DPI in mice (Figure 1). Likewise, as a result of verum (local and distal) EA stimulation, the rodent instinctive burrowing activity was largely increased (Figure 2). In addition, local EA was shown to provide better analgesia outcomes than distal EA over 14 days only with mechanical withdrawal thresholds but not with burrowing assessment, which is consistent with previous literature [15]. A possible explanation for the slightly higher analgesic effect evoked by local EA is that acupoints per se exert different physiological effects depending on its location [26,27]. Furthermore, we hypothesize that local acupuncture inhibited the dental noxious stimuli avoiding a consecutive trigeminal neural signaling, which can be explained by the gate control theory [16,19]. Therefore, there is a need for a new approach into the acupoint selection underlying the treatment for a better understanding of it therapeutic benefits [28]. 

### 3.2. Neuroinflammatory Profile after Pulp Exposure

Convincing evidence supports the involvement of microglia in neuropathic and persistent orofacial pain [21]. In an experimental model of inferior alveolar and mental nerve injuries, microglial activation was observed for 14 continuous days, with evidence of delayed astrocytic activation [29]. In our study, we observed microglial and astrocytes activation in the Vc on days 21 and 28 in a time-dependent manner, similar to previous research that has reported that DPI markedly increases microglial activation in the Vc, beginning at 14 days and peaking at 21 days [11]. Moreover, previous literature has reported that DPI-induced activation of satellite glial cells in the trigeminal ganglion between days 7 and 28 [30]. In our study, despite the recovery in pain-related behaviors between days 14 and 21, there was glial activation in Vc at 21 days and continued up to day 28 after DPI (Figure 3). The activated microglia and astrocytes release numerous signaling molecules, including pro-inflammatory cytokines such as TNF-α, IL-1β, and IL-6 that play crucial roles in pain states [31]. After neuropathic pain, the production of IL-1β is enhanced in glial cells and neurons. IL-1β is key cytokine and plays an essential role in the central sensitization and hyperalgesia [32]. The upregulation of TNF-α expression induced by an intra-temporomandibular joint CFA (Complete Freund’s Adjuvant) injection in the Vc has been reported [33]. In our study, we observed high production of TNF-α, IL-1β, and IL-6 in response to pulp exposure, on day 21 after DPI. Although we have not examined if this enhancement in the production of cytokines is time-dependent, it is consistent with the peak activation phase of microglial cells and astrocytes. Furthermore, in a constriction injury to the infraorbital nerve in rats, astrocyte activation was not associated with overexpression of IL-6 mRNA in the Vc [34]. We hypothesize the discrepancy with our results, in which we noted high levels of IL-6, is due to the fact that the model differs markedly from the DPI model and also the rodent’s cavity was left open to the oral microbiota. It is probable that similar to periodontitis, the microorganisms in the dental plaque biofilm enter the systemic circulation, or the host response increases levels of proinflammatory cytokines in the CNS as previously reported [35,36]. Our study is the first to report long-term increases in levels of TNF-α, IL-1β and IL-6 in the Vc in a DPI Type III model (Figure 5).

### 3.3. Verum EA and Ibuprofen Prevents Neuroinflammation

Previous data have suggested that mechanisms involving microglia activation in somatic and trigeminal pain may share similitudes [37]. The study of glial cell alterations may provide new alternatives for disease management involving persistent pain, as is the case of EA in a DPI mice model. Several studies have reported the neuroprotective effect of acupuncture treatment may be partially mediated by inhibition of inflammatory factors and microglial activation following neuropathic pain [38,39]. For instance, an animal model of spinal cord injury (SCI), EA has shown neuroprotective effects by reducing levels of microglial and proinflammatory mediators, such as precursor pro-nerve growth factor and proinflammatory cytokines (TNF-α, IL-1β, and IL-6) [38]. Similarly, our study demonstrated that verum (local and distal) EA could inhibit DPI-induced microglial and astrocytes activation and reversed the production of TNF-α, IL-1β, and IL-6, which regulate synaptic plasticity (Figure 4 and Figure 5). Although we have not made the comparison, we did not observe a significant difference between local and distal EA, as the literature on this aspect has not yet been fully explored and the mechanisms in which one’s acupuncture provides neuroprotection still remain poorly understood [40]. On the other hand, the mechanism of action of NSAIDs in this case of ibuprofen also involves the peripheral prostaglandin synthesis inhibition, which is mediated by endogenous opioid peptides within the CNS [41,42]. Furthermore, after SCI, chronic ibuprofen administration does not exert neuroprotection [43]. In the present study, however, we observed that ibuprofen inhibited the activation/production of microglia, astrocytes and proinflammatory cytokines (TNF-α, IL-1β, and IL-6) and these findings are in line with previous literature that reported the beneficial effect of ibuprofen [44,45].

### 3.4. Possible Mechanisms of the Analgesic and Neuroprotective Effects of Acupuncture

EA and manual acupuncture (MA) are the most frequently used acupuncture modalities. While MA may stimuli all types of afferent fibers (Aβ, Aδ, and C), EA can excite Aβ and Aδ-fibers through multiple possible pathways including the ascending facilitatory pathway in which one, the signal produced by the peripheral afferent fibers may reduce central sensitization modulated by neurotransmitters. Moreover, in the descending pain inhibitory pathway, the opioid peptides and their receptors play a crucial role in mediating acupuncture analgesia [15,46]. Interestingly, the low frequency of EA releases enkephalins, β-endorphin, and endomorphin to activate the opioid μ and δ receptors, while the high frequency of electroacupuncture releases dynorphin to activate the opioid κ receptor [47]. A recent study indicates that the cannabinoid CB1 receptor in the periaqueductal grey (PAG) is also involved as a possible pathway in the electroacupuncture analgesia [48]. In addition, EA is associated with anti-inflammatory effects in the periphery. Two possible pathways are indicated for this effect: the activation of sympathetic nerve fibers to intensify the displacement of opioid-containing cells and the reduction of COX-2 activity in the hypothalamus–pituitary–adrenal axis, a major neuroendocrine system, which consecutively reduces the production of prostaglandin [49]. Furthermore, increasing evidence suggests that neuropathic pain leads in microglia activation through ATP receptors [50]. In turn, activated microglia release ATP and cytokines, triggering astrocyte activation and contributing to neuroinflammation [51].

In summary, acupuncture may activate multiple signaling pathways to promote analgesia. Our results showed that verum (local and distal) EA dramatically decreased the levels of Iba1, GFAP, and cytokines, in the Vc. We assume that EA inhibits neuroinflammation, induced by DPI, mainly because of its analgesic effect. The observation that ibuprofen relieved DPI-induced pain and neuroinflammation supported our contention. However, the detailed mechanisms of acupuncture involved in cytokine expression and glial activation following DPI remain to be determined. 

## 4. Methods

### 4.1. Animals

Male ICR mice (25–30 g; BioLasco Taiwan Co., Ltd., Taipei, Taiwan) were housed under a 12:12 light/dark cycle with food and water available ad libitum in our animal facility for at least 4 days before the experiments, which were conducted between 10:00 and 18:00 h. The experimental procedure was approved by the China Medical University Institutional Animal Care and Use Committee, in accordance with the Care and Use of the Laboratory Animal Guidebook issued by the Chinese Taipei Society of Laboratory Animal Sciences (CMUIACUC-2017-168, 26/12/2016). 

### 4.2. Selection of DPI Models

Dental injury models are categorized as follows: Type 1: Mild injury with no pulp loss; Type II: Intermediate injury with partial pulp loss; Type III: Irreversible pulpitis characterized by advancing necrosis and systemic defense [6]. The Type III DPI model was selected for this study. The animals were deeply anesthetized with an intraperitoneal (i.p.) injection of chloral hydrate (0.40 mL/100 g body weight). The occlusal surface of the left first maxillary molar was drilled using an air-cooled, high-speed handpiece and a new #36 round bur. A deep cavity exposed the pulp, which was visually confirmed by an endodontic file #6. The tooth cavity remained open for 14, 21 or 28 days. All animals underwent the same dental procedure, except for the control group, which received anesthesia only.

### 4.3. Electroacupuncture (EA) Procedures

EA groups were placed in an induction chamber for 15 min, with the oxygen flow meter adjusted to approximately 2.0 L/min. For each 20-min acupuncture session, a mask was connected to a non-rebreathing circuit (Bain) and the flow meter was adjusted to 0.4–0.8 L/min. Acupuncture needles were inserted into specific acupoints and electrical stimulation was applied by an Ito Trio-300 stimulator (Ito, Japan) at an intensity of 2 mA at 2 Hz, using a 150 μs pulse width. The needles differed by size: 32-gauge for the local and sham acupoints and 36-gauge for the distal acupoints. 

### 4.4. Verum (Local and Distal) and Sham EA Groups

Each study group involved between 6 and 9 mice. Eleven sessions of EA were given to each mouse, starting one day (1 d) after DPI then on days 2, 3, 5, 7, 9, 11, 13, 15, 17 and 21. All EA sessions were delivered on the same side (in the left forelimb) as the dental injury. The murine-equivalents of the human Daying (ST5) and Jiache (ST6) acupoints served as local acupoints; ST5 is located on the face, anterior to the angle of the mandible, in the depression anterior to the masseter attachment, while ST6 is located on the muscle belly, anterior–superior side of the mandibular angle [52]. The murine-equivalents of the human Hegu (LI4) and Quchi (LI11) acupoints served as distal acupoints. The murine LI4 is located on the first dorsal interossei, radial to the midpoint of the second metacarpal bone in the forelimb, while LI11 is located in the depression at the lateral end of the cubital crease in the right forelimb [52]. As in previous research, the sham acupoints were located in the middle of the lateral deltoid muscle, in a region distant from most meridians [27]. The selection of acupoints was based on two of a total of three principles that are used commonly by practitioners indicating that: local acupoints selection (near the symptoms area), distant acupoints selection (along the meridian) and also based on symptom differentiation [28]. Likewise, the mice trans-positional acupoint system was followed, as previously described [52], (Figure S1).

### 4.5. Ibuprofen Group

Ibuprofen (Sigma-Aldrich, St. Louis, MO, USA) was administered by oral gavage at a dose of 30 mg/kg, 30 min prior to behavioral testing, as per previously described procedures [53]. The initial ibuprofen dose was given starting one day (1 d) after DPI then on days 2, 3, 5, 7, 9, 11, 13, 15, 17 and 21, following the same schedule as the EA treatment. Controls received an oral dose of drinking water.

### 4.6. Von Frey Testing

Mice were placed in cages for habituation for 1 h before undergoing testing with an electronic von Frey instrument (EVF-3; Bioseb, France), as previously reported [54]. The experimenter held the mice during testing and the filament was applied perpendicularly to the orofacial area on the DPI side (between the whisker pad and eye) with sufficient force to cause withdrawal [24]. Responses were considered positive when we observed a brisk head withdrawal, or vocalization/crying [55]. Negative responses as rapid unilateral or bilateral forepaw swipe down the snout or attempt to attack the filament and were not considered in the study. For each mouse, the pain threshold was calculated as the average of 3 measurements recorded within a 3 seconds interval, following previously described procedures [34]. All animals underwent baseline testing one day before the DPI procedure, then again on days 1, 3, 7, 14 and 21. 

### 4.7. Burrowing Behavioral Test

The burrowing behavioral test has proven sensitivity for measuring general “well-being” of rodents; any disturbance in normal healthy conditions (e.g., peripheral nerve injury or inflammation-associated pain) is reflected by reductions in spontaneous burrowing behavior, such as a decrease in the innate tendency of the mice to spontaneously displace items from tubes within their home cage [56]. We constructed and filled burrows with 200 g of food pellets, placed the mice in the home cage and weighed the number of pellets displaced within each 2-h session, as per previously described methods [25,56]. All mice underwent baseline measurements and then repeat testing sessions on days 1, 3, 7, 14 and 21.

### 4.8. Immunohistochemistry

At the end of the experiments, each mouse was deeply anesthetized with urethane 10% and then transcardially perfused with saline followed by 4% paraformaldehyde (PFA) fixative, as per previously described procedures [57,58]. Brain sections of 30 μm were cut in the coronal plane with a cryostat at −20 °C and processed for immunohistochemistry (IHC) analysis. The sections were placed in PBS pH 7.4 at 4 °C and stored for later use. The nonspecific binding sites in sections were blocked with bovine serum albumin and incubated overnight with an antibody against ionized calcium-binding adaptor molecule-1 (polyclonal rabbit anti-Iba1; 1:400 dilutions; Wako Chemicals USA, Richmond, VA, USA) and antibody against glial fibrillary acidic protein (polyclonal mouse anti-GFAP; 1:300 dilutions; Wako Chemicals USA, Richmond, VA, USA). Sections were incubated in rabbit or mouse polyclonal to anti-goat immunoglobulin G secondary antibody (1:300; Vector Laboratories, TPE, TWN) to visualize the staining. Sections were developed in DAB (3,3′-diaminobenzidine) solution for 5 min and washed 3 times for 10 min with Trisbuffered saline. Iba1^+^ and GFAP^+^ cell bodies were scanned with a NanoZoomer-XR digital slide scanner (Hamamatsu, Hamamatsu City, Japan) and processed by its viewing platform (NDP.view2).

### 4.9. Western Blot

Proteins from the Vc brainstem area were extracted at 21 days from all mice, as per previously described procedures [59]. The samples were loaded on precast 8% or 12% SDS-PAGE gels using approximately 20 μg protein in each lane. Membranes were blocked with 5% BSA in tris-buffered saline (TBS) for 1 h at room temperature and incubated overnight at 4 °C with a primary antibody: goat anti-Iba-1 (1:2000; #ab5076; Abcam), mouse anti-GFAP (1:2000; #3670; CST), rabbit anti-TNF-α (1:2000; ab9739; Abcam), rabbit anti-IL-1β (1:2000; ab9722; Abcam) and rabbit anti IL-6 (1:20,000; ab229381; Abcam) signals were normalized to β-actin (1:10,000; GTX629630) as an internal control. The film signals were digitally scanned and quantified using FUSION FX software.

### 4.10. Statistical Analyses

All values are expressed as the mean ± standard error of the mean (S.E.M.). All statistical analyses were performed using GraphPad Prism software. For behavioral investigations, between-group differences were calculated by one-way ANOVA and Dunnett’s post hoc test. In addition, one-way ANOVA followed by Tukey’s post hoc test was used for Supplementary data. For Western blot analysis, between-group differences were analyzed by the Student’s *t*-test. A *p*-value of <0.05 was considered statistically significant.

## 5. Conclusions

We provided the evidence for the first time that verum (local and distal) EA and ibuprofen treatment may inhibit microglia and astrocytes activation and suppress the production of major proinflammatory cytokines in a DPI model. Our findings provide significantly novel insights into the pathology and potential alternative treatment modality for dental pain.

## Supplementary Materials

Supplementary materials can be found at https://www.mdpi.com/1422-0067/21/7/2628/s1. Figure S1. Image showing acupoint locations used for EA treatment. Table S1. Von-Frey test statistical parameters derived from the one-way ANOVA followed by Tukey’s post hoc test. Table S2. Burrowing test statistical parameters derived from the one-way ANOVA followed by Tukey’s post hoc test.
