# Supplementary material for: Analgesic and Neuroprotective Effects of Electroacupuncture in a Dental Pulp Injury Model—A Basic Research
Source: Int J Mol Sci. 2020 Apr 9;21(7):2628. doi: 10.3390/ijms21072628 (PMC7178196; doi:10.3390/ijms21072628)
Supplement: Supplementary file 1 [file ijms-21-02628-s001.pdf]

Supplementary Data

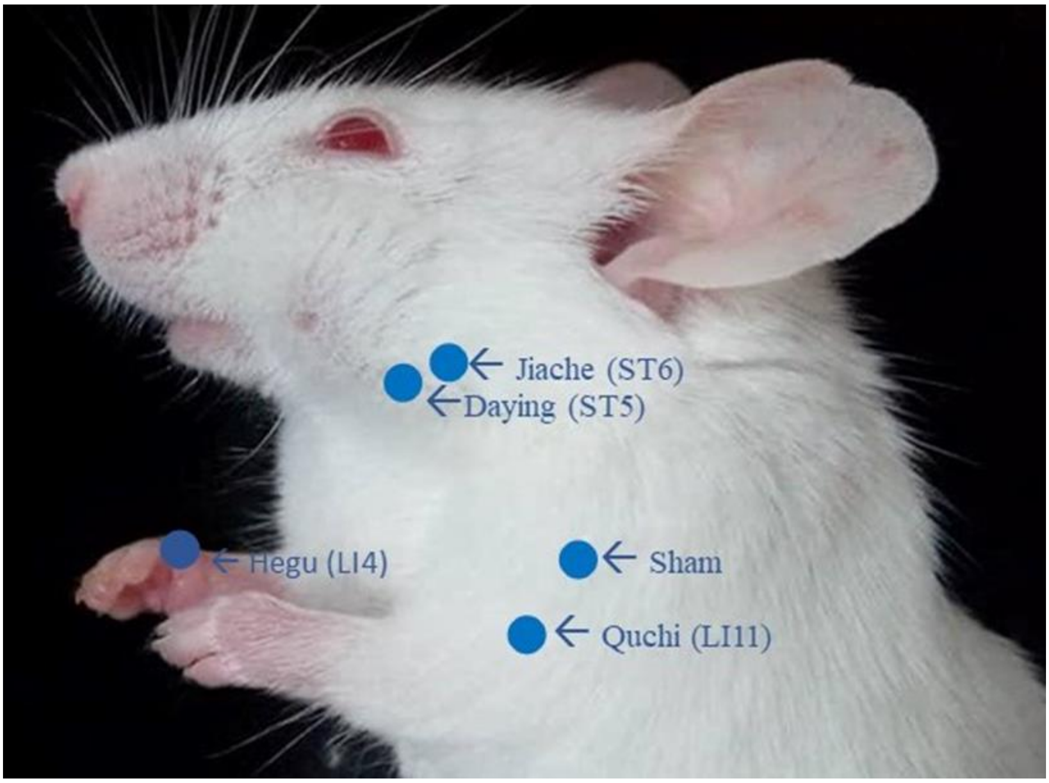

Figure S1. Image showing acupoint locations used for EA treatment.

The murine equivalents of the human acupoints: Daying (ST5) and Jiache (ST6) acupoints served as local acupoints and Hegu (LI4) and Quchi (LI11) acupoints served as distal acupoints. Sham acupoints were located in the middle of the lateral deltoid muscle.

Table S1. Von-Frey test statistical parameters derived from the one-way ANOVA followed by Tukey's post hoc test.

Von-Frey test	Comparison Groups	P value *P*	Symbols denoted		Comparison Groups	P value *P*	Symbols denoted
**Baseline**	Control vs DPI	No	ns	**Day-01**	Control vs DPI	Yes	***
	Control vs LI	No	ns		Control vs LI	Yes	***
	Control vs ST	No	ns		Control vs ST	Yes	***
	Control vs Sham	No	ns		Control vs Sham	Yes	***
	Control vs Ibu	No	ns		Control vs Ibu	Yes	***
	DPI vs LI	No	ns		DPI vs LI	No	ns
	DPI vs ST	No	ns		DPI vs ST	Yes	**
	DPI vs Sham	No	ns		DPI vs Sham	No	ns
	DPI vs Ibu	No	ns		DPI vs Ibu	Yes	*
	LI vs ST	No	ns		LI vs ST	No	ns
	LI vs Sham	No	ns		LI vs Sham	No	ns
	LI vs Ibu	No	ns		LI vs Ibu	No	ns
	ST vs Sham	No	ns		ST vs Sham	No	ns
	ST vs Ibu	No	ns		ST vs Ibu	No	ns
	Sham vs Ibu	No	ns		Sham vs Ibu	No	ns
**Day-03**	Control vs DPI	Yes	***	**Day-07**	Control vs DPI	Yes	***
	Control vs LI	Yes	***		Control vs LI	Yes	***
	Control vs ST	Yes	***		Control vs ST	Yes	***
	Control vs Sham	Yes	***		Control vs Sham	Yes	***
	Control vs Ibu	Yes	***		Control vs Ibu	Yes	***
	DPI vs LI	Yes	*		DPI vs LI	Yes	*
	DPI vs ST	Yes	***		DPI vs ST	Yes	*
	DPI vs Sham	No	ns		DPI vs Sham	No	ns
	DPI vs Ibu	Yes	***		DPI vs Ibu	No	ns
	LI vs ST	No	ns		LI vs ST	No	ns
	LI vs Sham	Yes	*		LI vs Sham	No	ns
	LI vs Ibu	No	ns		LI vs Ibu	No	ns
	ST vs Sham	Yes	***		ST vs Sham	Yes	*
	ST vs Ibu	No	ns		ST vs Ibu	No	ns
	Sham vs Ibu	Yes	***		Sham vs Ibu	No	ns

**Von-Frey test**	**Comparison Groups**	**P value *P***	**Symbols denoted**		**Comparison Groups**	**P value *P***	**Symbols denoted**
**Day-14**	Control vs DPI	Yes	***	**Day-21**	Control vs DPI	No	ns
	Control vs LI	Yes	**		Control vs LI	No	ns
	Control vs ST	Yes	*		Control vs ST	No	ns
	Control vs Sham	Yes	***		Control vs Sham	No	ns
	Control vs Ibu	Yes	***		Control vs Ibu	No	ns
	DPI vs LI	No	ns		DPI vs LI	No	ns
	DPI vs ST	Yes	*		DPI vs ST	No	ns
	DPI vs Sham	No	ns		DPI vs Sham	No	ns
	DPI vs Ibu	No	ns		DPI vs Ibu	No	ns
	LI vs ST	No	ns		LI vs ST	No	ns
	LI vs Sham	No	ns		LI vs Sham	No	ns
	LI vs Ibu	No	ns		LI vs Ibu	No	ns
	ST vs Sham	Yes	*		ST vs Sham	No	ns
	ST vs Ibu	No	ns		ST vs Ibu	No	ns
	Sham vs Ibu	No	ns		Sham vs Ibu	No	ns

Table S2. Burrowing test statistical parameters derived from the one-way ANOVA followed by Tukey's post hoc test.

Burrowing test	Comparison Groups	P value *P*	Symbols denoted		Comparison Groups	P value *P*	Symbols denoted
**Baseline**	Control vs DPI	No	ns	**Day-01**	Control vs DPI	Yes	***
	Control vs LI	No	ns		Control vs LI	Yes	***
	Control vs ST	No	ns		Control vs ST	Yes	***
	Control vs Sham	No	ns		Control vs Sham	Yes	***
	Control vs Ibu	No	ns		Control vs Ibu	No	ns
	DPI vs LI	No	ns		DPI vs LI	No	ns
	DPI vs ST	No	ns		DPI vs ST	Yes	**
	DPI vs Sham	No	ns		DPI vs Sham	No	ns
	DPI vs Ibu	No	ns		DPI vs Ibu	Yes	***
	LI vs ST	No	ns		LI vs ST	No	ns
	LI vs Sham	No	ns		LI vs Sham	No	ns
	LI vs Ibu	No	ns		LI vs Ibu	Yes	***
	ST vs Sham	No	ns		ST vs Sham	Yes	***
	ST vs Ibu	No	ns		ST vs Ibu	Yes	***
	Sham vs Ibu	No	ns		Sham vs Ibu	Yes	***
**Day-03**	Control vs DPI	Yes	***	**Day-07**	Control vs DPI	Yes	***
	Control vs LI	Yes	***		Control vs LI	Yes	***
	Control vs ST	Yes	***		Control vs ST	Yes	***
	Control vs Sham	Yes	***		Control vs Sham	Yes	***
	Control vs Ibu	Yes	*		Control vs Ibu	Yes	*
	DPI vs LI	Yes	**		DPI vs LI	Yes	**
	DPI vs ST	Yes	***		DPI vs ST	Yes	***
	DPI vs Sham	No	ns		DPI vs Sham	No	ns
	DPI vs Ibu	Yes	***		DPI vs Ibu	Yes	***
	LI vs ST	No	ns		LI vs ST	No	ns
	LI vs Sham	Yes	*		LI vs Sham	Yes	*
	LI vs Ibu	Yes	***		LI vs Ibu	Yes	***
	ST vs Sham	Yes	***		ST vs Sham	Yes	***
	ST vs Ibu	Yes	**		ST vs Ibu	Yes	**
	Sham vs Ibu	Yes	***		Sham vs Ibu	Yes	***

**Burrowing test test**	**Comparison Groups**	**P value *P***	**Symbols denoted**		**Comparison Groups**	**P value *P***	**Symbols denoted**
**Day-14**	Control vs DPI	Yes	***	**Day-21**	Control vs DPI	No	ns
	Control vs LI	No	ns		Control vs LI	No	ns
	Control vs ST	No	ns		Control vs ST	No	ns
	Control vs Sham	Yes	***		Control vs Sham	No	ns
	Control vs Ibu	No	ns		control vs Ibu	No	ns
	DPI vs LI	Yes	*		DPI vs LI	No	ns
	DPI vs ST	Yes	**		DPI vs ST	No	ns
	DPI vs Sham	No	ns		DPI vs Sham	No	ns
	DPI vs Ibu	Yes	**		DPI vs Ibu	No	ns
	LI vs ST	No	ns		LI vs ST	No	ns
	LI vs Sham	Yes	*		LI vs Sham	No	ns
	LI vs Ibu	No	ns		LI vs Ibu	No	ns
	ST vs Sham	Yes	**		ST vs Sham	No	ns
	ST vs Ibu	No	ns		ST vs Ibu	No	ns
	Sham vs Ibu	Yes	**		Sham vs Ibu	No	ns
